# Novel epigenetic network biomarkers for early detection of esophageal cancer

**DOI:** 10.1186/s13148-022-01243-5

**Published:** 2022-02-14

**Authors:** Alok K. Maity, Timothy C. Stone, Vanessa Ward, Amy P. Webster, Zhen Yang, Aine Hogan, Hazel McBain, Margaraet Duku, Kai Man Alexander Ho, Paul Wolfson, David G. Graham, SPIT Study Group, Stephan Beck, Andrew E. Teschendorff, Laurence B. Lovat

**Affiliations:** 1 CAS Key Lab of Computational Biology, Shanghai Institute for Nutrition and Health, University of Chinese Academy of Sciences, Chinese Academy of Sciences, 320 Yue Yang Road, Shanghai, 200031 China; 2 Division of Surgery and Interventional Science, University College London, Gower Street, London, WC1E 6BT UK; 3 University of Exeter Medical School, University of Exeter, Exeter, UK; 4 Key Laboratory of Medical Epigenetics and Metabolism, Institutes of Biomedical Sciences, Fudan University, Shanghai, 200032 China; 5 Division of GI Services, University College London Hospitals NHS Foundation Trust, 235 Euston Road, London, NW1 2BU UK; 6 UCL Cancer Institute, University College London, Gower Street, London, WC1E 6BT UK

**Keywords:** Esophageal adenocarcinoma, DNA methylation, Saliva, Barrett’s esophagus, Single-cell RNA-Seq, Biological networks

## Abstract

**Background:**

Early detection of esophageal cancer is critical to improve survival. Whilst studies have identified biomarkers, their interpretation and validity is often confounded by cell-type heterogeneity.

**Results:**

Here we applied systems-epigenomic and cell-type deconvolution algorithms to a discovery set encompassing RNA-Seq and DNA methylation data from esophageal adenocarcinoma (EAC) patients and matched normal-adjacent tissue, in order to identify robust biomarkers, free from the confounding effect posed by cell-type heterogeneity. We identify 12 gene-modules that are epigenetically deregulated in EAC, and are able to validate all 12 modules in 4 independent EAC cohorts. We demonstrate that the epigenetic deregulation is present in the epithelial compartment of EAC-tissue. Using single-cell RNA-Seq data we show that one of these modules, a proto-cadherin module centered around CTNND2, is inactivated in Barrett’s Esophagus, a precursor lesion to EAC. By measuring DNA methylation in saliva from EAC cases and controls, we identify a chemokine module centered around CCL20, whose methylation patterns in saliva correlate with EAC status.

**Conclusions:**

Given our observations that a CCL20 chemokine network is overactivated in EAC tissue and saliva from EAC patients, and that in independent studies CCL20 has been found to be overactivated in EAC tissue infected with the bacterium *F. nucleatum*, a bacterium that normally inhabits the oral cavity, our results highlight the possibility of using DNAm measurements in saliva as a proxy for changes occurring in the esophageal epithelium. Both the CTNND2/CCL20 modules represent novel promising network biomarkers for EAC that merit further investigation.

**Supplementary Information:**

The online version contains supplementary material available at 10.1186/s13148-022-01243-5.

## Background

One of the most urgent needs in the clinical management of esophageal cancer is a reliable test for early detection of disease, which would help significantly towards improving what are currently dismal 5-year survival rates [1–3]. Given the low compliance to undergo endoscopy, an early detection test would ideally also be non-invasive and cheap enough to implement for routine screening. While many studies have identified promising epigenetic biomarkers for esophageal cancer [4–9], a key drawback hampering biological interpretation and successful validation is cell-type heterogeneity of analyzed tissues [10–13]. Cell-type heterogeneity refers to the presence of stromal cells, notably immune cells and fibroblasts, in addition to the resident epithelial cells of the tissue, with variations in the corresponding cell-type fractions generally accounting for most of the data-variance when analyzing DNA methylation or gene-expression [10]. Thus, it is important to adjust for such cell-type heterogeneity when inferring cancer biomarkers.

Here we aimed to identify robust biomarkers for esophageal adenocarcinoma (EAC) by using state-of-the-art computational methodology to address the challenges posed by cell-type heterogeneity and multiple-testing. Specifically, we improve robustness and the false positive rate by not searching for individual differentially altered genes, but by searching for gene-modules that are jointly differentially methylated and differentially expressed, using our previously validated FEM (Functional Epigenetic Modules) algorithm to identify such modules in the context of a high quality protein–protein-interaction (PPI) network [14, 15]. A number of such integrative module detection algorithms have since emerged [16, 17], the rationale being that DNAm changes in disease often accompany gene expression changes that map to specific biological pathways and functional modules [15]. This systems-approach can therefore remove false positives and ensure the likelihood of discovering true positives, despite the crude nature of the underlying PPI networks. Because of the confounding effect posed by cell-type heterogeneity, we here extend FEM, by combining it with HEpiDISH [18], an algorithm designed to perform cell-type deconvolution of complex epithelial tissues. Briefly, we apply HEpiDISH to estimate total epithelial, immune and fibroblast fractions in the EAC samples from the TCGA cohort [19], and which are subsequently used to infer statistics of differential DNA methylation and mRNA expression reflecting associations with EAC that are not driven by underlying changes in cell-type composition. Following identification of FEM-modules associated with EAC, we perform extensive validation in independent EAC cohorts, and finally explore the potential utility of specific modules for early detection of EAC. We do this in the context of a single-cell RNA-Seq dataset comprising cells from Barrett’s esophagus, a premalignant lesion that precedes EAC development. Our findings also lead us to explore the inferred gene-modules in the context of DNAm in saliva, an easily accessible tissue that contains a significant proportion of squamous epithelial cells [18, 20–22], which may serve as a suitable surrogate for recording DNAm changes in the cells that give rise to Barrett’s esophagus and EAC.

## Results

### Identification of epigenetically deregulated gene-modules in EAC

**Fig. 1:**
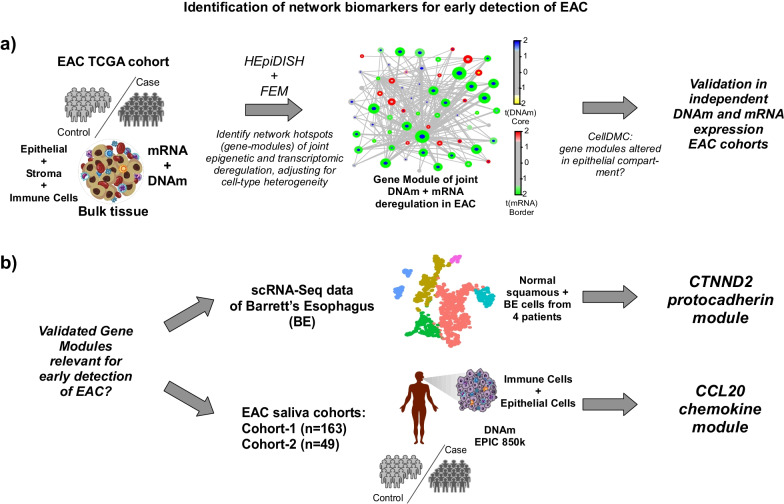
Identification of network biomarkers in EAC. **a** Using the TCGA DNA methylation and mRNA expression datasets for EAC, we identify gene-modules of joint epigenetic and expression deregulation in EAC compared to normal-adjacent tissue, using our Functional Epigenetic Modules (FEM) algorithm whilst adjusting for stromal heterogeneity. The latter is accomplished by estimating total epithelial, total immune cell and total fibroblast fractions in the TCGA samples using our HEpiDISH algorithm. FEM searches for gene-modules in the context of a PPI network. Subsequently, we apply our CellDMC algorithm to ascertain if the DNA methylation changes underlying the inferred gene modules are happening in the epithelial compartment of the tissue. Finally, we validate inferred modules in independent DNA methylation and mRNA expression EAC datasets. **b** Modules validating in (**a**) are then explored for their potential utility as early detection markers. This is done in two ways. In one case we analyse scRNA-Seq data from Barrett’s and normal esophagus to explore if any modules are deregulated in Barrett’s. In the second case, we explore if promising gene-modules exhibit variable DNA methylation patterns in saliva from cohorts containing both EAC and healthy subjects, where we also apply cell-type deconvolution methods to estimate epithelial fractions in saliva

**Fig. 2:**
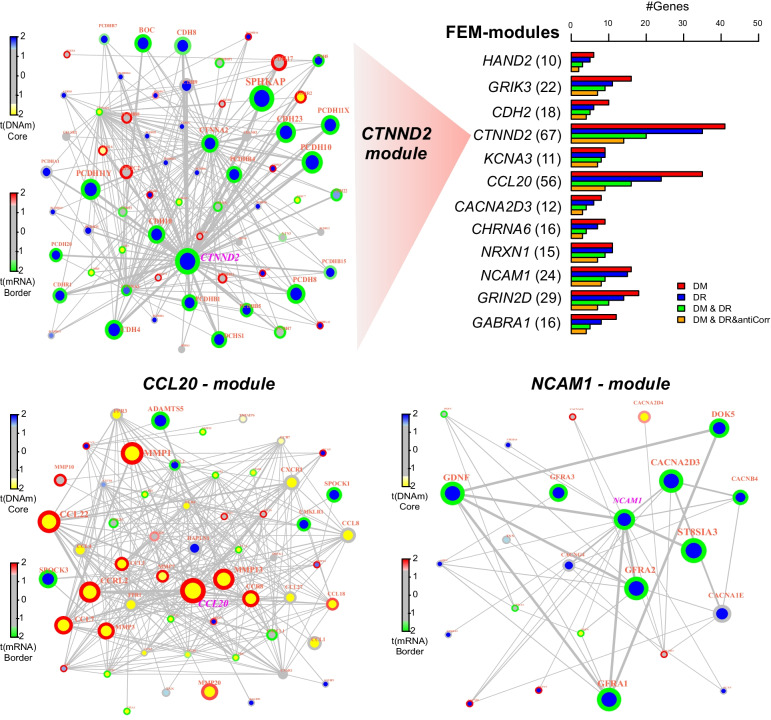
FEM-modules derived from TCGA EAC cohort. Barplot lists the significant FEM-modules labeled by the seed-gene with the number of genes in each module given in brackets. Length of bars indicate the number of genes within the module that are significantly differentially methylated (DM), significantly differentially expressed (DR), jointly differentially methylated and differentially expressed (DM & DR) and the subset of these that exhibit anticorrelation between promoter DNAm and gene-expression. We display 3 modules defined by seed-genes, *CTNND2, CCL20* and *NCAM1*. The node color indicates differential DNAm, with the border color indicating differential expression, as specified in the color-bars

The overall strategy to detect biomarkers for early detection of esophageal cancer was largely driven by power considerations and availability of appropriate datasets (Fig. 1). The underlying idea behind our strategy was to leverage the higher effect sizes and larger number of samples of EAC-tissue cohorts to identify EAC-biomarkers (Fig. 1a), and subsequently to filter these for potential relevance in early detection, either in preneoplastic esophageal tissue or in suitable surrogate tissues like saliva (Fig. 1b). In order to identify robust biomarkers associated with EAC, we combined a systems-epigenomics algorithm called FEM (Functional Epigenetic Modules) [14, 15] with a cell-type deconvolution algorithm called HEpiDISH [18], applying them both in an integrative fashion to the TCGA EAC cohort [19] (Fig. 1a). This cohort has genome-wide DNAm (12 normal-adjacent + 50 EAC) and RNA-Seq (8 normal-adjacent + 79 EAC) data available, allowing us to identify joint DNAm and mRNA expression changes in a predominantly stage T1-3 EACs cohort (approximately 20% of the tumors are T1, 34% T2, 41% T3 and only 5% T4). By using FEM [14], we search for hotspots (gene-modules) of joint differential DNAm and mRNA expression between EAC and normal-adjacent tissue in the context of a protein–protein-interaction (PPI) network (Fig. 1a), a strategy that maximizes the chance of discovering true positive associations [23] and which we have previously and successfully applied to other cancer-types [15]. However, because normal and cancer tissue represent admixtures of esophageal epithelial cells with immune and other stromal cells (e.g. fibroblasts), we here extended the FEM algorithm to adjust for stromal heterogeneity when computing differential DNAm and mRNA expression statistics (Methods). In more detail, we used our HEpiDISH framework [18] to estimate total epithelial, total fibroblast and total immune cell fractions in each of the esophageal TCGA samples (Additional file 1: Fig. S1). SVD-analysis on the DNAm dataset revealed that the top principal component correlated most strongly with normal-cancer status (Additional file 1: Fig. S1). Cell-type fractions correlated strongly with lower ranked components but marginally also with PC1. Hence, when identifying differentially methylated genes (DMGs) and differential expressed genes (DEGs) between normal and cancer tissue, we used the estimated cell-type fractions as covariates in the linear regression models, so as to avoid confounding by potential changes in cell-type composition. Using this strategy, we identified a total of 12 gene-modules (Fig. 2, Additional file 1: Table S1, Additional file 1: Fig. S2), centered around 12 marker “seed” genes, which included *CTNND2*, *CCL20* and *NCAM1*. For instance, the module around *CTNND2* revealed widespread promoter hypermethylation and downregulation of many proto-cadherin genes, including *CTNND2* itself (Fig. 2). In contrast, the *CCL20* module was dominated by promoter hypomethylation and overexpression, suggesting activation of this chemokine-network in EAC (Fig. 2). For each module we computed a “FEM-activation score” (Methods) reflecting the degree of deregulation, which was generally speaking independent of tumor-stage (Additional file 1: Fig. S3). Despite some of the genes in these modules (e.g. *CTNND2*, *CDH18*, *CCL20*) displaying copy number variation (CNV) in esophageal cancer [19], we verified that the statistics of differential DNAm and mRNA expression for all gene module members were very robust upon adjustment for CNV-status (Additional file 1: Figs. S4, S5), thus demonstrating that CNV is not a confounder.

### Validation of epigenetically deregulated gene-modules in independent cohorts

**Fig. 3:**
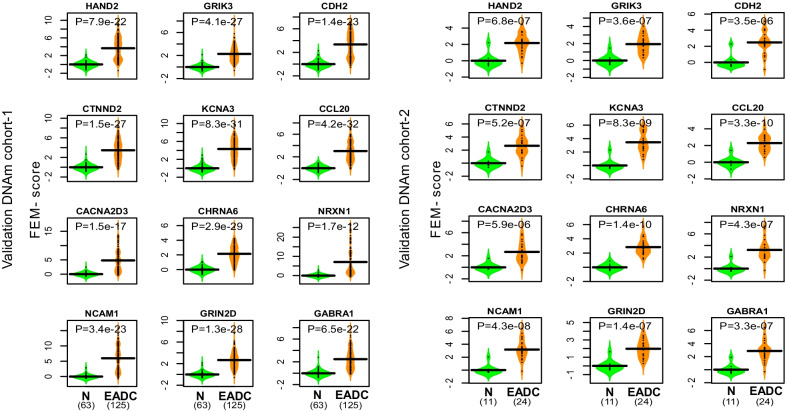
Validation of our EAC modules in independent DNAm cohorts. Violin plots comparing predicted FEM-scores for each of the 12 EAC modules in each of two independent DNAm EAC cohorts. Validation cohort-1 is from GSE72872. Validation cohort-2 is from GSE89181. *P* values are from a one-tailed Wilcoxon rank sum test

To ascertain the biological significance of the 12 gene-modules, we collated a total of 4 independent EAC cohorts, 2 profiling mRNA expression and 2 profiling DNAm (Additional file 1: Table S2). For each module in each dataset, we computed a score (FEM-score), assessing the level of epigenetic or transcriptomic deregulation of each sample, and asked if the FEM-scores can discriminate normal from cancer tissue (Methods). In the case of DNAm we were able to validate all 12 modules with high statistical significance in both cohorts (Fig. 3). In the case of mRNA expression, the majority of modules were validated in at least one of the two cohorts (Additional file 1: Figs. S6–S9). Thus, this demonstrates that FEM has indeed identified bona-fide epigenetic and transcriptomic markers of EAC.

### Deregulation of FEM-modules occurs in the epithelial compartment

**Fig. 4:**
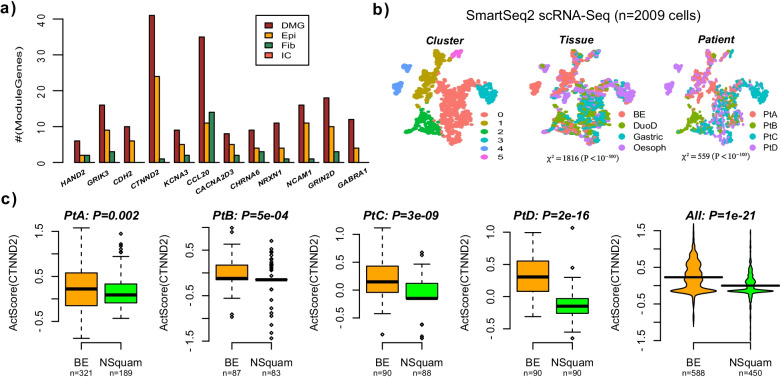
The CTNND2 module is altered in the epithelial cells of EAC and Barrett’s Esophagus. **a** Barplot displaying the number of differentially methylated genes (DMG), and the subset of these that are specifically differentially methylated in the epithelial (Epi), fibroblast (Fib) and immune cells (IC) for each of the 12 EAC FEM-modules, as determined in the EAC TCGA DNAm dataset. **b** tSNE diagrams for the 2009 epithelial single cells profiled in samples derived from 4 patients diagnosed with BE. The first panel displays the inferred 6 clusters. The middle panel labels the cells by the site of sampling (tissue): BE = Barrett’s Esophagus, DuoD = duodenum, Gastric = gastroesophageal junction, Oesoph = adjacent normal squamous cells from oesophagus. The right panel labels the cells by the patient they derived from. The Chi-Square statistics and associated *P* values were derived from a contingency table test, assessing how unevenly tissue and patient are distributed among the inferred clusters. **c** Violin plots displaying the FEM cancer-score for the CTNND2 module in a scRNA-Seq dataset comparing epithelial cells from Barrett’s Esophagus lesions (BE) to adjacent normal squamous epithelial cells for each of 4 different patients diagnosed with BE. Last panel is for the case where all cells from all patients are merged. *P* values are from a one-tailed Wilcoxon rank sum test

Next, we wanted to assess in which cell-types the cancer-associated molecular alterations underlying our FEM-modules occur. To this end, we applied CellDMC [24], an algorithm designed to detect cell-type specific DNAm changes to the discovery EAC DNAm dataset from the TCGA (Methods), in order to identify cell-type specific differentially methylated genes (cts-DMGs). For most FEM-modules, about half of the significantly differentially methylated genes within a module were predicted to be cts-DMGs, and by far these were mostly epithelial cts-DMGs (Fig. 4a). For instance, 24 of the 41 DMGs in the *CTNND2*-module were epithelial cts-DMGs, with only 1 being specific to the fibroblast compartment and none being specific to the immune-cell compartment. Only the *CCL20*-module exhibited an equal number of cts-DMGs in the epithelial and fibroblast compartments.

### The CTNND2 module is deregulated in the epithelial cells of Barrett’s Esophagus

To ascertain that our modules are capturing molecular changes in the epithelial cells of the esophagus, and to also explore the timing of the molecular alterations, we analyzed a Smart-Seq2 single-cell RNA-Seq dataset derived from 4 patients with Barrett’s esophagus (BE) at each of 4 sites, including normal squamous epithelial cells as well as epithelial cells derived from BE, duodenum and stomach [25] (Methods). Indeed, we reasoned that some of our FEM-modules may already exhibit alterations in the epithelial cells from BE, which would make them more attractive for early detection strategies. Using the Seurat pipeline (Methods) we inferred a total of 6 clusters, with tissue/site and patient distributed unevenly across these clusters, but with the tissue/site dominating the source of variation and clustering (Fig. 4b). Next, we computed the FEM-score of our modules in each of the single cells (Methods). Unfortunately, due to the relatively high dropout rate, we could only compute the FEM-score for the *CTNND2* module, since only for this module did we find enough gene members with variable expression across the 1038 cells (588 BE & 450 normal-squamous). However, in each of the 4 patients the calculated FEM-score of the *CTNND2* module was higher in the epithelial cells from BE compared to the adjacent normal squamous epithelium, and results were highly significant when merging the cells from all 4 patients together (Fig. 4c). Thus, these data indicate that the molecular alterations underlying the CTTND2-module is present in the esophageal epithelium as an early field-defect in BE.

### Association of CCL20 module with EAC in saliva

Next, we assessed the 12 FEM-modules in two cohorts of saliva specimens (Cohorts 1 and 2) from subjects representing 4 different stages in EAC development, including normal (*N*, *n*(Cohort1) = 65, *n*(Cohort2) = 5), nondysplastic BE (NDBE: *n*(1) = 33, *n*(2) = 15), high grade dysplasia (HGD: *n*(1) = 14, *n*(2) = 15) and cancer (C: *n*(1) = 51, *n*(2) = 14) (Methods). Illumina DNAm profiles were generated for these cohorts using the EPIC platform that measures over 850,000 CpGs. The rationale for testing these modules in saliva at the DNAm level is that saliva contains a proportion of epithelial cells, likely derived from the buccal epithelium, which we posit may be informative of the DNAm changes seen in BE, HGD and EAC tissue.

**Fig. 5:**
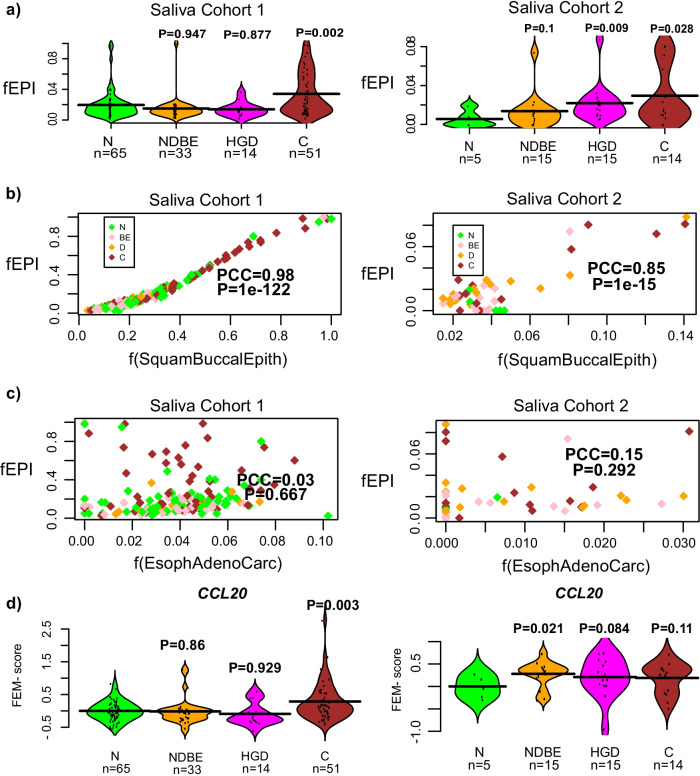
Correlation of CCL20 module with EAC status in saliva. **a** Violin plots displaying the total epithelial fraction (fEPI, y-axis), as estimated using HEpiDISH with our Epithelial-Fibroblast-ImmuneCell DNAm reference, against disease stage (x-axis) in the two saliva cohorts, as indicated. *P* values derive from a one-tailed Wilcoxon rank sum test, comparing each disease stage to the normal-state. N = normal, NDBE = non-dysplastic Barrett’s Esophagus, HGD = high grade dysplasia, C = Esophageal Adenocarcinoma. **b** Scatterplot of the estimated total epithelial fraction (fEPI, y-axis) vs the estimated squamous buccal epithelium fraction (x-axis). Pearson correlation coefficient (PCC) and *P* value are given. Samples have been colored according to disease stage using same coloring scheme as in (**a**). **c** As (**b**), but now displaying the estimated esophageal adenoma carcinoma cell fraction along the x-axis. **d** Violin plots displaying the FEM EAC score (y-axis) versus disease stage (x-axis), for the CCL20-module in the two saliva cohorts. *P* values derive from a one-tailed Wilcoxon rank sum test, comparing each disease stage to the normal-state

To explore this, we first aimed to demonstrate that saliva contains a significant fraction of epithelial cells, and that these cells derive from the buccal epithelium of the oral cavity. We applied our HEpiDISH algorithm [18] to the two saliva cohorts in order to estimate the epithelial fraction. This confirmed that epithelial fractions were non-negligible, and interestingly, that they increased with cancer stage in both cohorts (Fig. 5a). To confirm the source of epithelial cells, we built a novel DNAm reference matrix with representative DNAm profiles for squamous buccal epithelium, EAC cell-lines and immune cells (Methods), and reapplied our HEpiDISH algorithm [18, 26] to infer corresponding cell-type fractions in our saliva samples. In both cohorts, this revealed an excellent correlation of the estimated buccal squamous epithelial content with the total epithelial fraction as determined with our previous DNAm reference matrix (Fig. 5b). In contrast, no correlations were observed between the total epithelial fraction and the fraction of esophageal adenocarcinoma cells (Fig. 5c). This confirms that the epithelial fraction in saliva derives from the adjacent squamous buccal epithelium. Finally, we computed the FEM-scores for all our modules in two saliva cohorts, which revealed a number of associations (Additional file 1: Figs. S10, S11). However, upon careful inspection most associations did not validate between Cohorts 1 and 2, with the exception of the *CCL20* module, which did reveal a consistent increase with disease stage in both cohorts (Fig. 5d). Specifically, the *CCL20* module displayed an AUC = 0.65 (*P* = 0.002) for discriminating normal from cancer in Cohort-1, and an AUC = 0.7 (*P* = 0.11) in Cohort-2, the non-significance in Cohort-2 attributable to the much smaller sample size of Cohort-2 (Fig. 5d, Additional file 1: Figs. S10, S11). Although these associations were partly driven by the increased epithelial fraction, the associations with EAC-status remained marginally significant under a linear regression model where we adjusted for the epithelial content (Cohort-1: *t* = 1.9, *P* = 0.06; Cohort-2: *t* = 1.8, *P* = 0.09). Thus, these data suggest that there are DNAm changes of the *CCL20* module in saliva of EAC-cases that “mimick” those seen in EAC-tissue.

## Discussion

Here we have identified two promising network biomarkers for EAC, one characterized by promoter hypermethylation and underexpression of a proto-cadherin gene module centered around *CTNND2*, and another module characterized by promoter hypomethylation and overexpression of a chemokine-network centered around *CCL20*. Unlike previous studies, we identified these biomarkers by adjusting for variations in the stromal compartment between normal and EAC-tissue, using state-of-the-art cell-type deconvolution methods, and furthermore demonstrating that the underlying DNAm chances are occurring primarily in the epithelial compartment of cells. In support of this we analysed scRNA-Seq data, demonstrating that the *CTNND2*-module is aberrantly deregulated in the epithelial cells derived from Barrett’s Esophagus in each of 4 separate BE patients. Thus, we have identified a potential field defect in BE that could be informative of disease progression. Admittedly, this association would be more relevant if demonstrated in BE patients with HGD, yet given that the *CTNND2* module is not only deregulated in BE but also in EAC, it is highly plausible that the *CTNND2* module would also be deregulated in intermediate stages such as BE + HGD lesions. Caution must be exercised however as our findings in BE are based on only 4 samples and results were derived from a scRNA-Seq data exhibiting over 90% sparsity. In any event, our data makes a strong case for scaling up scRNA-Seq studies to larger numbers of patients. While a number of previous studies have already indicated the potential importance of *CTNND2/WNT*-signaling in EAC [27], a potentially more striking observation is that deletion of *CTNND1* has been shown to lead to esophageal squamous cell carcinoma (ESCC) development [28, 29]. Thus, it would appear that *CTNND-*genes play key tumor suppressor roles in both types of esophageal cancer, with their inactivation being mediated by potentially different molecular mechanisms (promoter hypermethylation in the case of *CTNND2* and deletion in the case of *CTNND1*), which is possibly related to the different etiology of EAC vs ESCC.

*CCL20/*chemokine signaling has also been previously implicated in esophageal cancer [30]. Our observation that the *CCL20* module appears to be overactivated in both EAC tissue as well as in saliva from EAC patients is striking, because an independent study has shown *CCL20* to be strongly overexpressed in EAC-tissue displaying elevated levels of the bacterium *F. nucleatum* [31], a bacterium that also inhabits the oral cavity. It is therefore plausible that epigenetic changes associated with this bacterium in esophageal tissue could also be present in the buccal epithelial cells of the oral cavity. Of note, BE and EAC develop from metaplasia where squamous epithelial cells get replaced by columnar epithelium, thus the cell-of-origin of EAC is likely to be a basal progenitor squamous epithelial cell, similar to the cells in the buccal epithelium, thus providing further rationale as to why buccal epithelial cells may be an informative surrogate tissue of EAC. While we admit that this is hypothetical, our data clearly suggests an important future line of investigation: given that buccal swabs contain a much larger fraction of epithelial cells compared to saliva [18, 21], it would be interesting for future studies to jointly measure the microbiome and DNA methylome in such swabs, so as to explore the hypothesis that *CCL20* (and DNAm alterations occurring elsewhere in the genome) is epigenetically deregulated as a function of *F.nucleatum* levels. Given the relative ease-of-access of buccal swabs, this could provide a promising avenue for developing non-invasive screening strategies for esophageal cancer. There is also another reason why buccal swabs or saliva may be an appropriate tissue for assessing esophageal cancer risk. We have previously observed that DNAm changes that accrue in the buccal epithelium of smokers are very similar to those seen in a wide range of different epithelial cancer-types, including lung and esophagus [32]. It is likely that such common DNAm changes are driven by an increased cellular turnover and mitotic-rate associated with factors such as inflammation [33–35]. Inflammation could also be driven by bacterial infections, in addition to gastroesophageal reflux disease, the main risk factor for EAC. Thus, it is plausible that DNAm changes occurring in the buccal epithelium may record DNAm changes in other tissue-types that are also exposed to the same pathogen (*F.nucleatum*) or carcinogen (smoking).

Besides the sparsity and low number of clinical samples of the scRNA-Seq data, another major limitation of this study is the substantial inter-cohort and inter-individual variation of the epithelial fraction in saliva. These differences between individuals and cohorts likely stem from differences in sample collection and processing, but may also be influenced by confounders such as alcohol consumption, gingivitis or other medical conditions of the oral cavity. Of note, such high inter-individual variability has also been observed in buccal swabs [18, 20]. Thus, moving forward, future work will need to understand the sources of inter-individual variability in epithelial fractions within saliva and buccal swabs.

## Conclusions

In summary, by using innovative computational methods, we have here identified *CTNND2* and *CCL20* modules that represent two promising novel network biomarkers for early detection of EAC. We propose future studies to fully explore the clinical significance of these modules.

## Methods

### Normalization and preprocessing of tissue EAC datasets

#### TCGA EAC DNAm, mRNA expression and CNV dataset

We used the DNAm, mRNA expression and CNV datasets from the TCGA [19]. These datasets were normalized as described by us previously [36, 37]. Briefly, in the case of DNAm data, missing values in the level-3 data were imputed using impute.knn [38] with *k* = 5 and an imputation threshold of 30% (i.e. only probes with less than 30% missing values imputed, rest of probes were removed), followed by BMIQ normalization [39]. There were 12 normal-adjacent + 50 EAC samples measured with Illumina 450 k beadarrays, and 8 normal-adjacent + 79 EAC samples measured with RNA-Seq.

#### Validation bulk tissue DNAm datasets

- Krause et al. [40] is an Illumina 450 k DNAm dataset comprising genome-wide DNA methylation profiles for 250 samples including 125 EAC and 64 normal adjacent squamous samples. Data is available from GEO (http://www.ncbi.nlm.nih.gov/geo/ under accession number GSE72872). Raw idat files were processed with minfi [41]. Probes with SNPs or with more than 25% missing values were removed. Rest of probes were imputed with impute.knn [38] with k = 5. Type-2 probe bias was corrected using BMIQ [39]. One sample was removed due to low quality as assessed using BMIQ, resulting in 63 normal adjacent and 125 EAC samples.
- Kaz et al. [42] is an Illumina 450 k DNAm dataset comprising genome-wide DNA methylation profile of 127 esophageal samples, including 24 EAC and 11 normal adjacent squamous. Data is available from GEO (http://www.ncbi.nlm.nih.gov/geo/ under accession number GSE89181). Raw idat files were processed as described for Krause et al.

#### Validation bulk tissue gene expression datasets

- Krause et al. [40] performed Illumina gene expression (HumanHT-12 V4.0 bEAChip) profiling of 65 esophageal samples (48 EAC, 17 normal adjacent squamous). We downloaded the provided log-normalized dataset from GEO (http://www.ncbi.nlm.nih.gov/geo/ under accession number GSE72874) and performed inter-sample normalization using quantile normalization with limma R package [43].
- Lu et al. [44] used Affymetrix Human Gene 1.0 ST Arrays to profile 10 normal adjacent squamous and 12 EAC. Raw data was downloaded from GEO (http://www.ncbi.nlm.nih.gov/geo/under accession number GSE92396). We successively applied intra-sample and inter-sample normalization in the dataset using affy and limma R packages, respectively.

### scRNA-Seq dataset of Barrett’s Esophagus (BE)

We analysed a scRNA-Seq Smart-Seq2 dataset from Owen et al. [25] which profiled BE specimens as well as normal-adjacent tissue for a number of different patients. We downloaded the gene-count matrix from the website provided with the publication, and processed it with the Seurat pipeline [45]. We selected cells with at least 200 expressed genes, and selected genes expressed in at least 3 cells. Counts were then log-normalized using a scale factor of 10^4^. In total we obtained 2009 cells from 4 different patients and from 4 distinct tissues: normal esophagus (*n* = 450), duodenum (*n* = 349), gastric (*n* = 622) and BE (*n* = 588). To assess the sources of variability in this dataset we ran the standard Seurat pipeline, including variable feature selection (with variance stabilization), PCA, graph-based clustering and tSNE visualization. Graph-based clustering was done over 8 components (inferred with ElbowPlot function) and at a resolution of 0.1, which resulted in 6 clusters.

### Inference of FEM-modules from TCGA EAC dataset

To identify epigenetically deregulated gene-modules in EAC, we applied our Functional Epigenetic Module (FEM) algorithm [14]. Briefly, this approach integrates DNA methylation with gene expression data in the context of a protein–protein-interaction (PPI) network to identify hotspots (gene-modules) where there is substantial coordinated DNAm and gene expression changes. The algorithm consists of three steps: (1) quantify t-statistics of differential DNAm (DM) and gene expression (DE) between adjacent normal and EAC for each gene in the PPI network, (2) the edges of the network are weighted according to an integrated statistic for each gene defining the edge, where the integrated statistic per gene is obtained from the corresponding differential DNAm and differential expression statistics, and (3) inference of hotspots as subnetworks of particularly high modularity, i.e. subgraphs where the average weighted edge density is high compared to the rest of the network. The PPI network consists of 11,751 genes annotated to NCBI Entrez identifiers and is derived from the Pathway Commons resource [46, 47]. In step-(1), in order to arrive at a single statistic for DM, we summarize DNAm for each gene as the average over TSS200 probes (i.e. probes within 200 bp of the TSS). If such probes are not available, we use 1^st^ Exon probes instead, and if also not available then we use the average over TS1500 probes. This strategy is motivated by the fact that for these regions, there is generally an inverse relation between DNAm and gene expression, and the algorithm seeks to identify gene modules where this pattern is observed frequently. To derive the statistics for DM and DE we then use the limma R-package [43, 48, 49]. In step-(2), the integrated statistic per gene is then constructed as

$$t_{g}^{\left( I \right)} = \left\{ {H\left( {t_{g}^{{\left( {DM} \right)}} } \right)H\left( { - t_{g}^{{\left( {DE} \right)}} } \right) + H\left( { - t_{g}^{{\left( {DM} \right)}} } \right)H\left( {t_{g}^{{\left( {DE} \right)}} } \right)} \right\}\left| {t_{g}^{{\left( {DM} \right)}} - t_{g}^{{\left( {DE} \right)}} } \right|$$

where *H(x)* is a Heaviside function, *H(x)* = 1 if *x* > 0 and *H(x)* = 0 if x < 0. If genes *g* and *h* are connected in the PPI network, we then assign the edge weight by taking the average of the corresponding integrated statistics of the genes, i.e.

$$w_{gh} = 0.5*\left( {t_{g}^{\left( I \right)} + t_{h}^{\left( I \right)} } \right)$$

To infer the modules (“FEM-modules”) in step-3, we then use a local greedy adaptation of a powerful spin-glass algorithm [23, 50]. The spin-glass algorithm tries to minimize the following Hamiltonian energy function

$$H\left( {\left\{ s \right\}} \right) = - \mathop \sum \limits_{g \ne h} \left\{ {w_{gh} - \gamma p_{gh} } \right\}\delta \left( {s_{g} ,s_{h} } \right)$$

where $s_{g}$ is the spin-state (i.e., module) the gene *g* belongs to, $\delta \left( {x,y} \right) = 1$ iff *x* = *y* and 0 otherwise, and $p_{gh}{ \sim}w_{g.} w_{h.}$ is the null probability (once normalized) with $w_{g.}$ denoting the weighted degree of gene *g.* In the local greedy version we try to grow modules around a number of “seed-genes” defined as the highest ranked genes by the integrated statistic $t_{g}^{\left( I \right)}$. We choose on the order of 100 seed genes, to ensure that most of the network is explored. In previous studies we have found that this number of seed genes works well and already leads to redundant modules. The spin-glass parameter $\gamma$ was chosen to be 0.5, as this parameter choice typically leads to modules in the size range of 10–100, which is the optimal size-range as shown by us previously [23]. The statistical significance of the modules is determined in two complementary ways: a module is grown from a given seed-gene in a deterministic fashion by adding the neighboring gene that minimizes the Hamiltonian energy and this process is continued until no further additions can decrease the energy function. This assesses the significance of the edge-weight density of the module in relation to the rest of the network and is strongly influenced by the topology of the network. As a second topology-independent test, we assess statistical significance of the inferred modules using a Monte Carlo (MC) randomization procedure (1000 Monte-Carlo runs), where we randomize the statistics over the network (thus keeping the topology fixed), recomputing modularity scores for each module and subsequently comparing the observed modularity to that of this empirically generated null distribution. The final FEM-modules are those with a *P* < 0.05.

### Computation of FEM-score in validation tissue EAC datasets

In the case of the DNAm validation sets, we first summarized the Illumina 850 k/450 k DNAm values to the promoters of the genes involved in a given module. This is done following our previously validated procedure [14]. This procedure averages the DNAm values of CpG probes mapping to within 200 bp of the TSS of each gene. If no probes map to within 200 bp, we use probes mapping to the 1st Exon, and if not available, we resort to probes mapping 1.5 kb upstream of the TSS. Subsequently, we select the genes in the module that were significantly differentially methylated in the original EAC TCGA cohort. For each of these genes, we then z-score normalize their DNAm profile over all samples within the given validation cohort, using the mean and standard deviation as estimated over the samples from normal/healthy individuals. That is, if $x_{gs}$ denotes the DNAm value for gene *g* in sample *s,* we compute

$$z_{gs} = \frac{{x_{gs} - \mu_{gN} }}{{\sigma_{gN} }}$$

where $\mu_{gN} , \sigma_{gN}$ denote the mean and standard deviation DNAm of gene *g* across the normal samples. The FEM-score for module *m* in sample *s* is then obtained as

$$FEMscore_{ms} = \frac{1}{\left| m \right|}\mathop \sum \limits_{g \in m} Sign\left( {t_{g} } \right) z_{gs}$$

where $\left| m \right|$ is the number of significantly differentially methylated genes in the module (as determined by the discovery TCGA EAC dataset) and $Sign\left( {t_{g} } \right)$ is the sign (+ 1/− 1) of the corresponding t-statistic of differential DNAm from the TCGA EAC cohort. Thus, the *FEMscore* assesses whether the deviation in DNAm relative to the normal state is consistent with the pattern observed in the TCGA EAC cohort, with a higher *FEMscore* in cancer patients indicating a coordinated epigenetic deregulation consistent with that seen in the discovery TCGA set.

In the case of the gene-expression datasets, the *FEMscore* of the module was computed by z-score normalizing the expression values of the module genes, using the mean and standard deviation of the gene over the normal samples, in direct analogy to the DNAm case. Here, only genes that were significantly differentially expressed in the discovery TCGA EAC cohort are used. Given the z-scores, the *FEMscore* is then obtained as the weighted average over all significant module genes, with the weight being + 1 if the gene is overexpressed in cancer relative to normal according to the TCGA dataset, and − 1 if underexpressed.

### Computation of FEM-score in scRNA-Seq data of BE

Here we selected cells from normal esophagus (*n* = 450) and BE (*n* = 588), and ran the above analysis to compute FEM-scores in each single cell, using the normal cells to define the z-score transformation. FEM-scores could only be reliably computed for the CTNND2-module: the number of significantly differentially expressed genes (from the TCGA EAC cohort) in each module that were also variable in the scRNA-Seq dataset was less than 5 for all modules except CTNND2 for which the number of variable genes was 11. FEM-scores were then compared between the normal and BE cells using Wilcoxon rank sum tests, stratified by patient.

### Computation of FEM-score in TCGA ESCA data

The procedure for computing the FEM-score in the TCGA data was slightly different from the previously described one, because for the TCGA we have both DNAm and RNA-Seq data. Briefly, the FEM-score for module *m* in sample *s* was computed as

$$FEMscore_{ms} = \frac{1}{\left| m \right|}\mathop \sum \limits_{g \in m} \left| {z_{gs}^{\left( M \right)} - z_{gs}^{\left( R \right)} } \right|$$

where $\left| m \right|$ is the number of significantly and consistently differentially methylated and differentially expressed genes in the module (as determined in the TCGA dataset itself), and where $z_{gs}^{\left( M \right)}$ and $z_{gs}^{\left( R \right)}$ are the corresponding z-scores for the DNAm and RNA-Seq datasets. By consistently differentially methylated and differentially expressed we mean that the pattern is anti-correlative (i.e., promoter hypermethylation and underexpression, or promoter hypomethylation and overexpression). The z-scores were computed as described previously, with the exception of the scores for the DNAm data where we added a constant offset term to the standard deviation, since the number of normal-adjacent samples with both DNAm and mRNA data is small (*n* = 6) and this can lead to spurious low variances and inflated scores in the DNAm-case. Specifically, for the DNAm-data we defined

$$z_{gs}^{\left( M \right)} = \frac{{x_{gs} - \mu_{gN} }}{{\sigma_{gN} + \gamma }}$$

where $\gamma$ was determined by requiring that the standard deviation of the *z-*scores for DNAm, as evaluated genome-wide over all genes in the experiment, equals the standard deviation of the *z*-scores for RNA-Seq data.

### Saliva sample collection

Saliva was collected from two groups of patients: the primary “training” cohort of 192 patients was collected through the SPIT Study (Saliva to predict risk of disease using transcriptomics and epigenetics, ISRCTN11921553) at 15 hospitals in the United Kingdom. This study was ethically approved by the West Midlands-Coventry and Warwickshire Research Ethics Committee (REC Reference No: 17/WM/0079). The validation cohort of 49 patients were collected through the BOOST Study (Barrett’s oesophagus surveillance with optical biopsy using spectroscopy and enhanced endoscopic imaging to target high-risk lesions, ISRCTN58235785) at a single hospital site (UCLH). This study was ethically approved by the London-Dulwich Research Ethics Committee (REC Reference No: 08/H0808/8). All participants gave written consent.

Patients were instructed to fast for a minimum of one hour and then to spit repeatedly into a saliva DNA collection device to a total of 2 mL. The primary cohort used Oragene DNA OG-500 (DNAGenotek, Ottawa, Canada) and the validation cohort used SimplOFy™ (Oasis Diagnostics® Corporation, Vancouver, Canada). Saliva was sent to the central laboratory at UCL and stored at − 80 °C until extraction.

DNA Extraction was carried out using the Zymo Quick-DNA™ Miniprep Plus Kit (Zymo Research, Irvine CA, USA). DNA quality was assessed using the Agilent Bioanalyser (Agilent Inc, Santa Clara, CA, USA). Only samples with a 260/280 nm ratio of > 1.5 were used. Bisulphite conversion was undertaken using the Zymo EZ-96 DNA methylation kit (Zymo Research, Irvine CA, USA) and samples containing 500 ng DNA were then DNAm profiled using the Illumina Infinium HumanMethylationEPIC BeadChip (Illumina San Diego, CA, USA).

### Analysis of saliva DNAm datasets

Raw signal intensities were processed from idat files through a standard pipeline that we have extensively validated [18]. Briefly, we processed idat files with the *minfi* R-package with no background correction and using the Illumina definition of beta-values. Probes with *P* values of detection < 0.05 were declared reliable measurements, the rest being set to NA. We removed cross-reactive probes, probes with more than 4 SNPs, probes with a SNP at the interrogated CpG, and probes with less than 90% coverage. Remaining NAs were imputed with the *impute* R-package using *impute.knn* function with *k* = 5 [38]. Type-2 probe bias was corrected using our BMIQ algorithm [39]. The final beta-valued data matrix for the discovery cohort contained 689,033 probes and 163 samples, of which 65 were from healthy controls, 33 from patients diagnosed with non-dysplastic Barrett’s Esophagus (NDBE), 14 with high-grade dysplasia (HGD) and 51 with EAC. In the case of the validation cohort, a total of 49 samples passed QC, of which 5 were normal, 15 NDBE, 15 HGD and 14 EAC.

### Computation of FEM-score in saliva EAC DNAm datasets

We applied the same procedure as for the tissue DNAm validation sets described above. Here, the normal samples used for performing the z-score transformation are the saliva samples from the age-matched healthy controls.

### Estimation of cell-type fractions in tissue and saliva DNAm datasets

In the case of the TCGA EAC cohort we estimated cell-type fractions from the Illumina 450 k DNAm data with our HEpiDISH algorithm [13, 18]. Briefly, this uses a DNAm reference matrix defined over 3 broad cell-types (total epithelial, total fibroblast and total immune-cell), in conjunction with Robust Partial Correlations (RPC) to yield estimates for the total epithelial, total fibroblast and total immune-cell fractions in each of the TCGA samples. In the case of the saliva DNAm datasets, the same procedure was applied to obtain total epithelial and total immune-cells fractions (the fibroblast fraction in saliva is negligible, which was also confirmed by HEpiDISH).

To assess the source of epithelial cells in saliva, we built a separate DNAm reference matrix defined over 3 main cell-types: squamous epithelial cells, epithelial cells representative of EAC cell-lines, and a generic immune-cell. In detail, the Illumina 450 k DNAm data from 48 purified immune-cells (6 × 8 cell-types) was derived from Reinius et al. [51]. From Iorio et al. [52] we obtained Illumina 450 k DNAm profiles for a total of 7 EAC cell-lines (ESO26, ESO51, FLO-1, KYAE-1, OACM5-1, OACP4C an SK-GT-4). For the squamous epithelial cells we first identified buccal swabs with over 95% purity, as determined with our HEpiDISH algorithm with the DNAm reference matrix defined above, and as applied to our large buccal swab 450 k DNAm dataset of 790 buccal swabs [32]. We only considered never-smokers to avoid DNAm changes induced by smoking. This resulted in 10 buccal swab samples of high epithelial purity, which we took as representative for putative squamous epithelial cells in saliva. To select the features (CpGs) defining our DNAm reference matrix, we performed differential DNAm analysis between the 48 IC, 7 EAC cell-line and 10 squamous epithelial cell samples using the limma empirical Bayes framework [49, 53]. Specifically, we performed 3 pairwise comparisons (IC vs. EAC + SqEpi), (SqEpi vs IC + EAC) and (EAC vs. IC + SqEpi). In all cases, we selected features with an FDR < 0.05 and a difference in mean DNAm greater than 0.9, except for the second comparison where we relaxed the threshold a little to 0.875. This ensured similar numbers of differentially methylated CpGs (DMCs): 136, 123 and 153 for the 3 comparisons, respectively. This resulted in a DNAm reference matrix defined over 412 unique CpGs and 3 cell-types (total IC, squamous epithelial, EAC). To estimate cell-type fractions in saliva, we then applied our HEpiDISH/RPC algorithm with this DNAm reference matrix.

## Supplementary Information

**Additional file 1.**  Contains all Supplementary Figures and Supplementary Tables.

## Data Availability

The discovery EAC tissue mRNA and DNAm datasets are derived from the TCGA and are freely available from https://portal.gdc.cancer.gov/. The 4 EAC datasets used for validation are available from GEO http://www.ncbi.nlm.nih.gov/geo/ under Accession Numbers GSE72874, GSE92396, GSE72872, GSE89181. The scRNA-Seq dataset from BE as well as the other Illumina 450 k DNAm datasets used here are all publicly available as specified in the respective Methods section and published references. The saliva Illumina DNA methylation datasets are only available on a collaborative basis by submitting a request to Prof. Laurence Lovat (l.lovat@ucl.ac.uk).

## Abbreviations

EAC: Esophageal adenocarcinoma
BE: Barrett’s esophagus
DMG: Differentially methylated gene
DMC: Differentially methylated CpG
FEM: Functional epigenetic modules
cts-DMG: Cell-type specific differentially methylated gene
FDR: False discovery rate
DNAm: DNA methylation
NDBE: Non-dysplastic Barrett’s esophagus
HGD: High-grade dysplasia
PPI: Protein–protein-interaction
TCGA: The Cancer Genome Atlas
CNV: Copy-number variation
