# Supplementary material for: Novel epigenetic network biomarkers for early detection of esophageal cancer
Source: Clin Epigenetics. 2022 Feb 14;14:23. doi: 10.1186/s13148-022-01243-5 (PMC8845366; doi:10.1186/s13148-022-01243-5)
Supplement: Supplementary file 1 — Additional file 1. Contains all Supplementary Figures and Supplementary Tables. [file 13148_2022_1243_MOESM1_ESM.pdf]

Supplementary Information for "Novel epigenetic network biomarkers for early detection of esophageal cancer"

SUPPLEMENTARY FIGURES

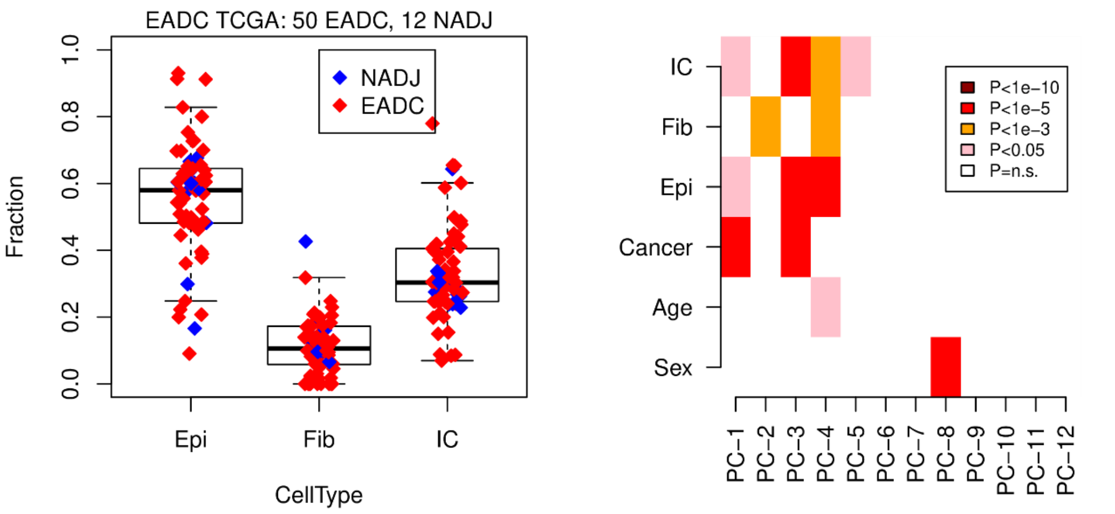

SI fig.S1: Estimation of cell-type fractions in esophageal adenocarcinoma TCGA cohort. Left panel is a boxplot displaying the estimated total epithelial (Epi), total fibroblast (Fib) and total immune cell (IC) fractions, with datapoints colored by normal-adjacent (NADJ) and EAC/EADC status. Right panel is a heatmap of correlation P-values between top PCs and various factors including the cell-type fractions, Cancer-status, Age and Sex.

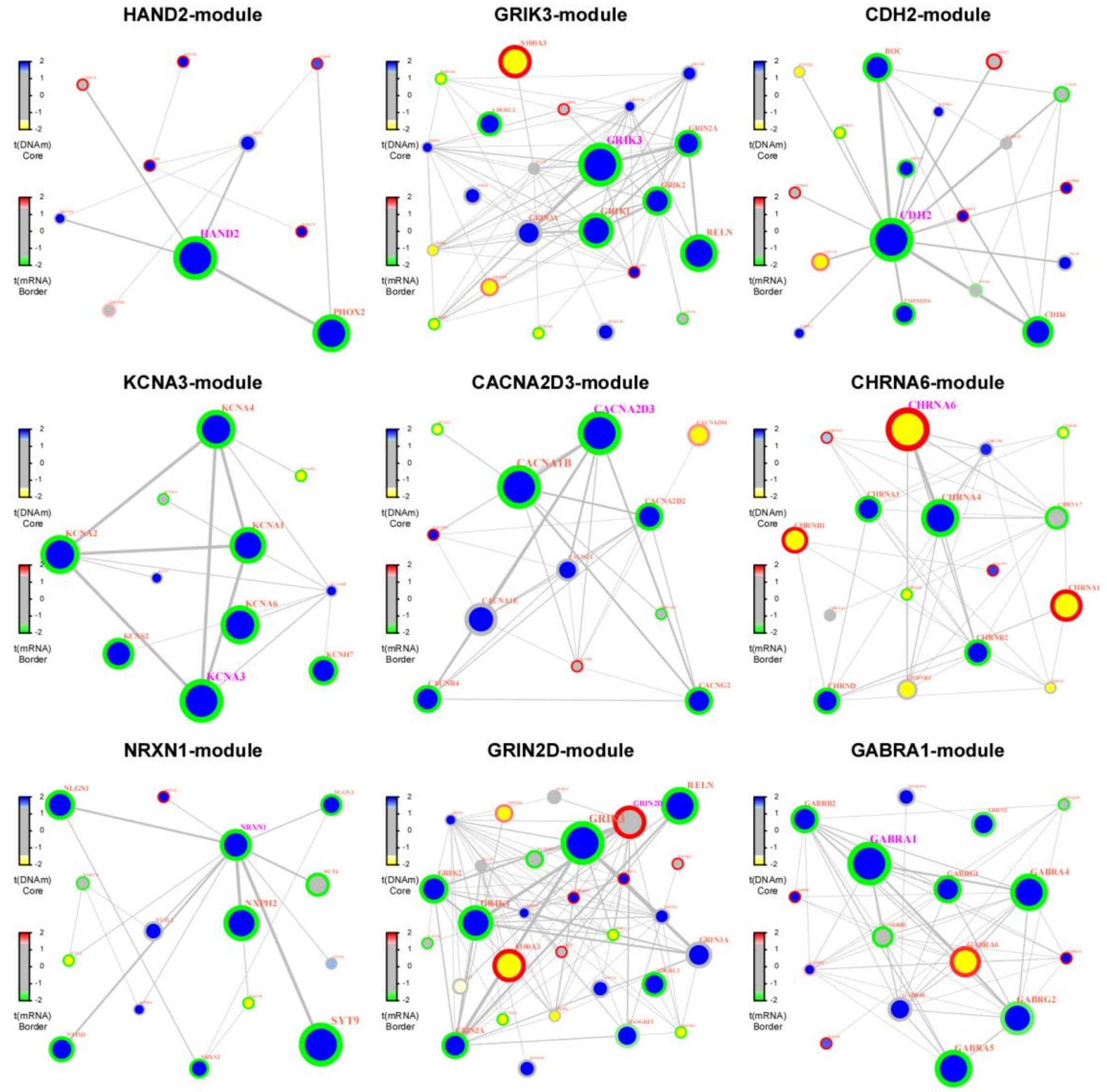

SI fig.S2: FEM-modules derived from EAC TCGA cohort. We display 9 of the 12 FEM-modules representing network hotspots of differential DNAm and mRNA expression in EAC compared to normal-adjacent tissue. Node colors indicate differential DNAm, border colors indicate differential expression, as indicated.

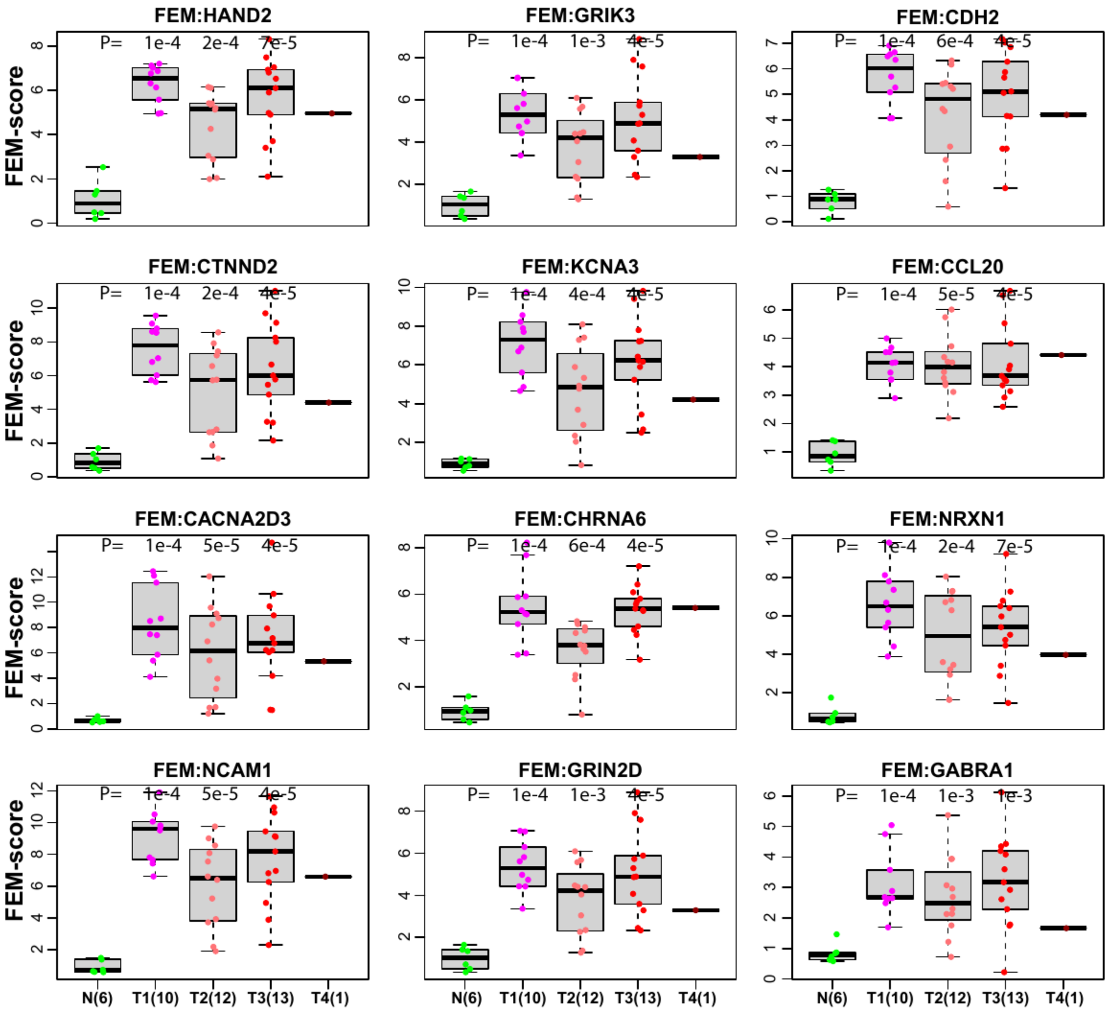

SI fig.S3: FEM-score patterns of modules across EAC-stages. For each of the 12 FEM modules, we display boxplots comparing the FEM-activation scores of each module across normal (N) and EAC stratified by stage (T1-T4). Number of samples in each group is given below each boxplot. The P-values are derived from a one-tailed Wilcoxon test comparing each group to N. For T4 we don't display P-value because only 1 sample was T4.

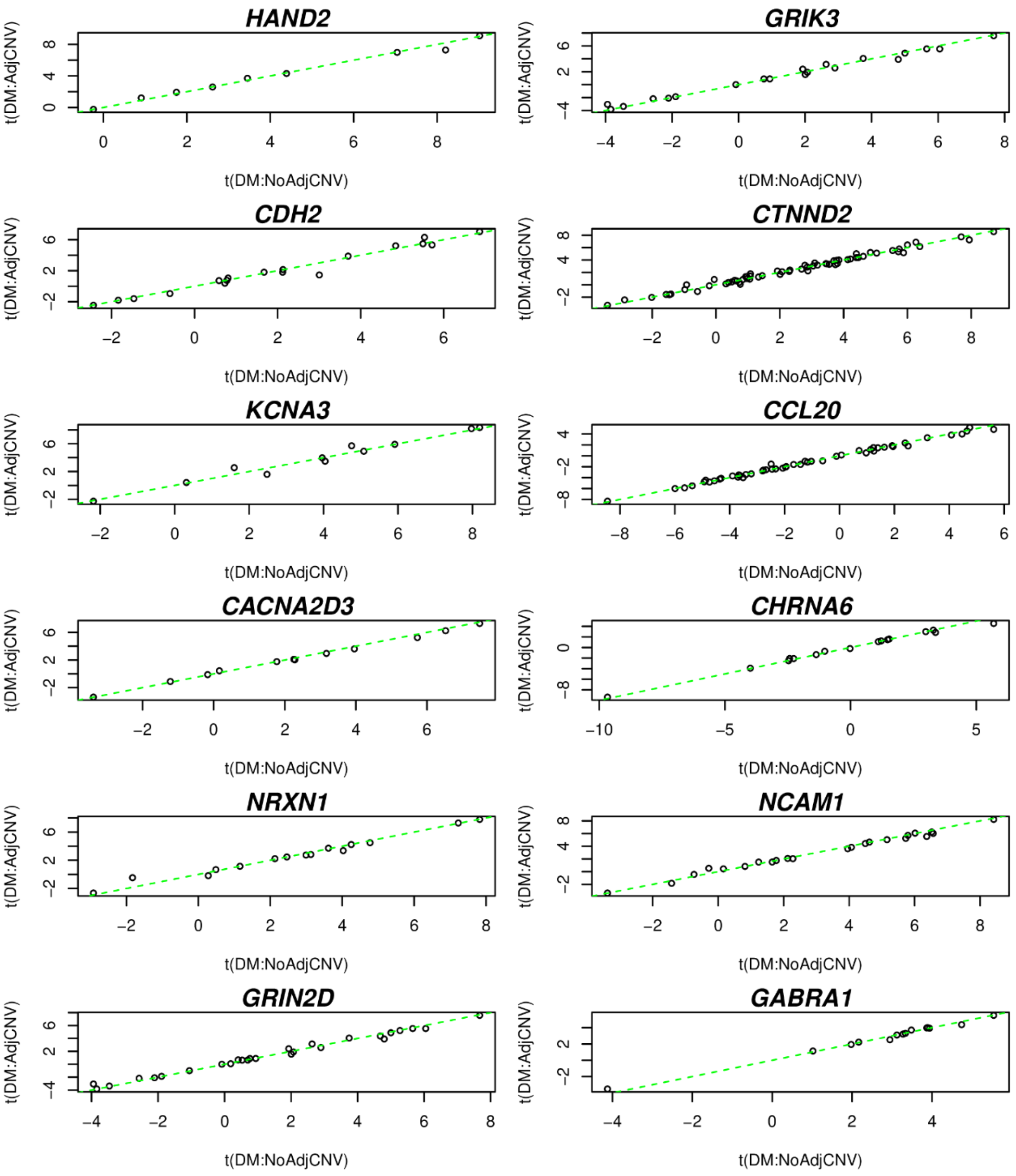

SI fig.S4: Robustness of differential DNAm statistics to CNV. For each of the 12 FEM modules, we display a scatterplot of the t-statistics of differential DNAm (DM) without adjustment for CNV (x-axis) vs the corresponding t-statistics upon adjustment for CNV (y-axis). Each data point represents a gene within the module. Green dashed line indicates the line of unit gradient (i.e. the line of a perfect Pearson Correlation of 1 and a RMSE of 0).

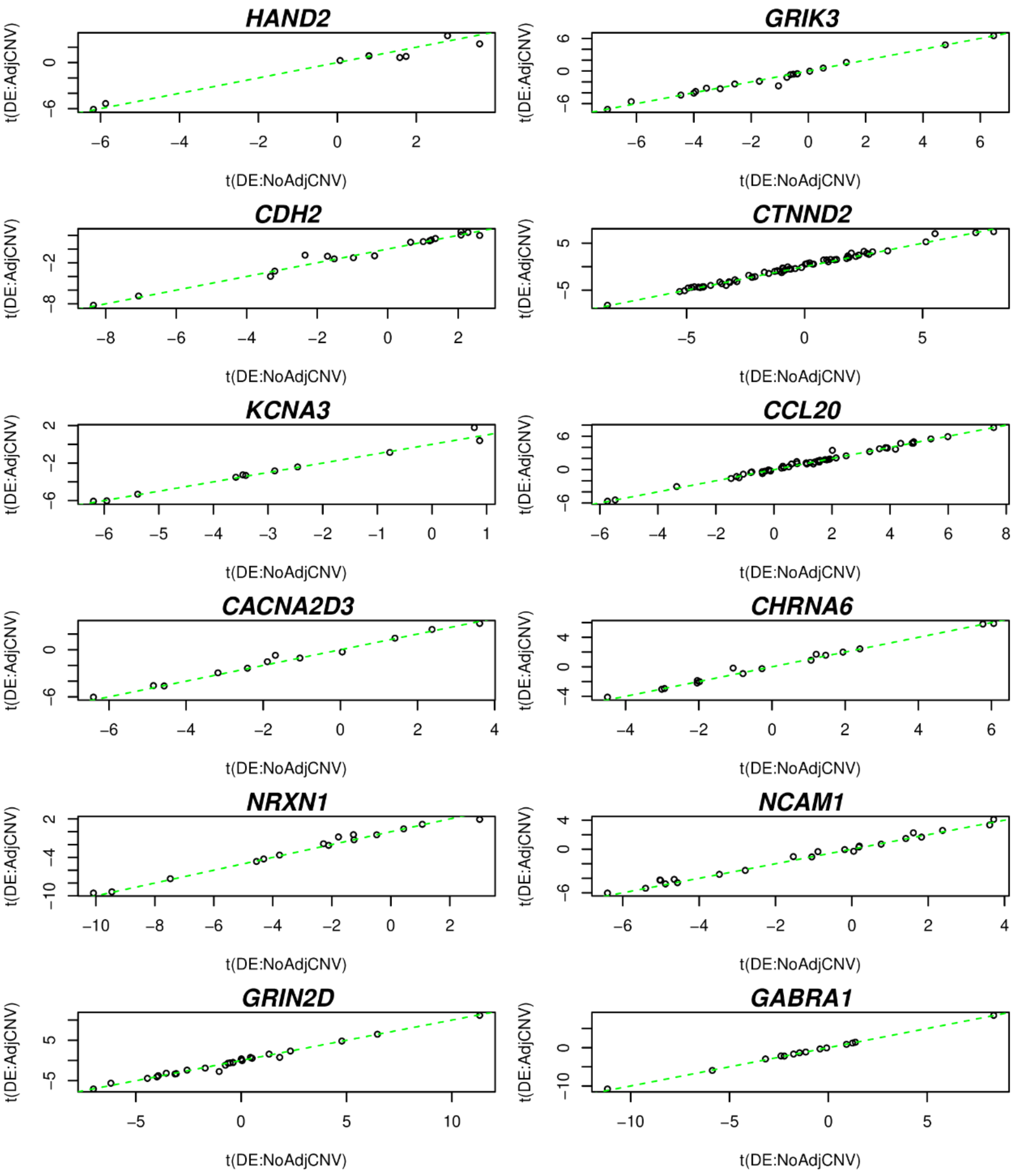

SI fig.S5: Robustness of differential mRNA statistics to CNV. For each of the 12 FEM modules, we display a scatterplot of the t-statistics of differential expression (DE) without adjustment for CNV (x-axis) vs the corresponding t-statistics upon adjustment for CNV (y-axis). Each data point represents a gene within the module. Green dashed line indicates the line of unit gradient (i.e. the line of a perfect Pearson Correlation of 1 and a RMSE of 0).

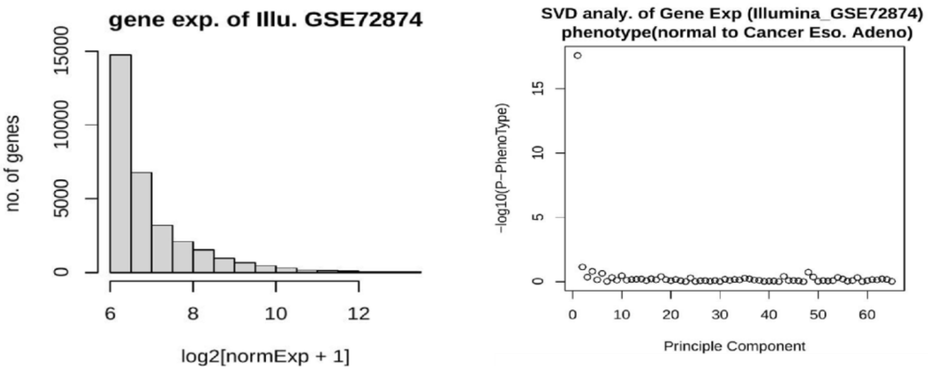

SI fig.S6: SVD analysis on GSE72874 gene-expression EAC dataset. Left panel: histogram of normalized gene-expression values. Right panel: Significance of correlation between top principal components and normal-cancer status.

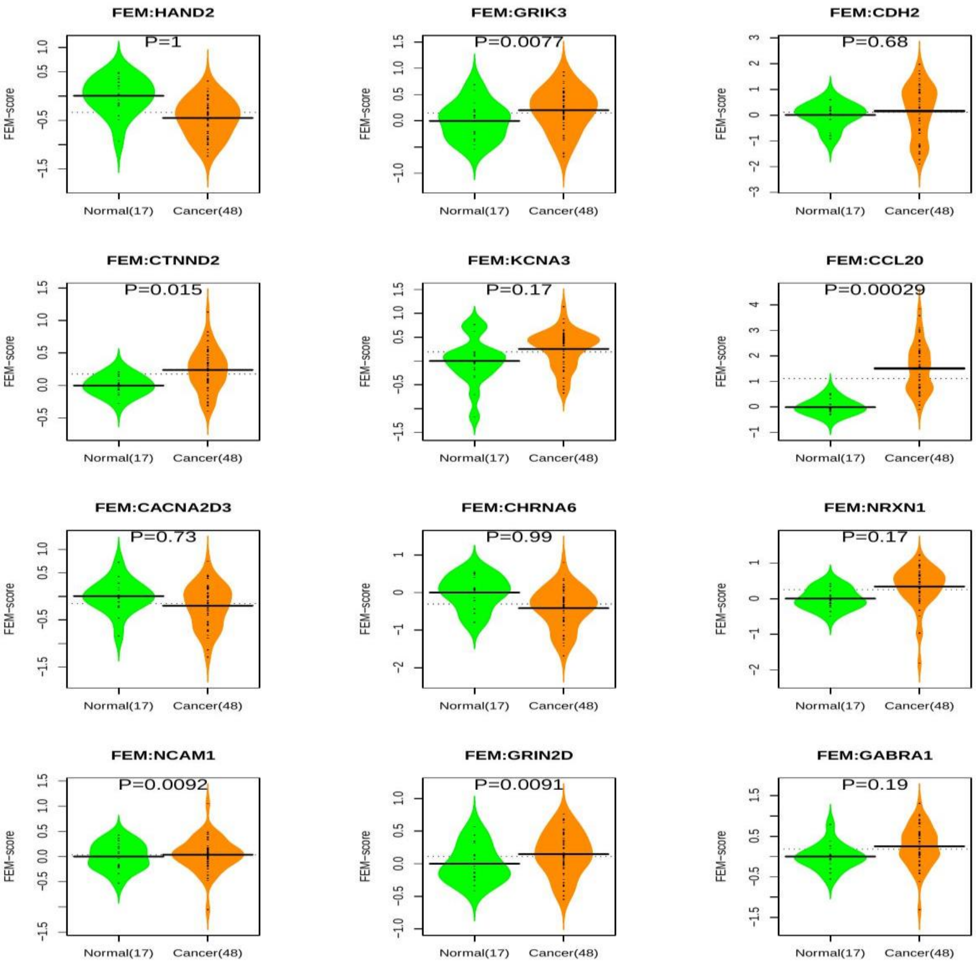

SI fig.S7: Association of FEM-scores in normal-cancer status in GSE72874 gene-expression dataset. Violin plots of FEM-scores for each of the 12 gene-modules. P-value is from a linear model of FEM-score against normal-cancer status with estimated IC cell fraction as covariate. Relative IC fraction was quantified using GSVA.

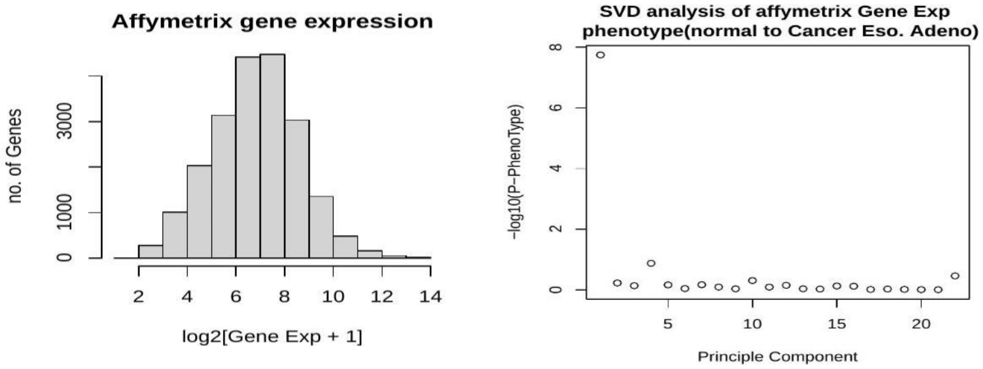

SI fig.S8: SVD analysis on GSE92396 Affymetrix gene-expression EAC dataset. Left panel: histogram of normalized gene-expression values. Right panel: Significance of correlation between top principal components and normal-cancer status.

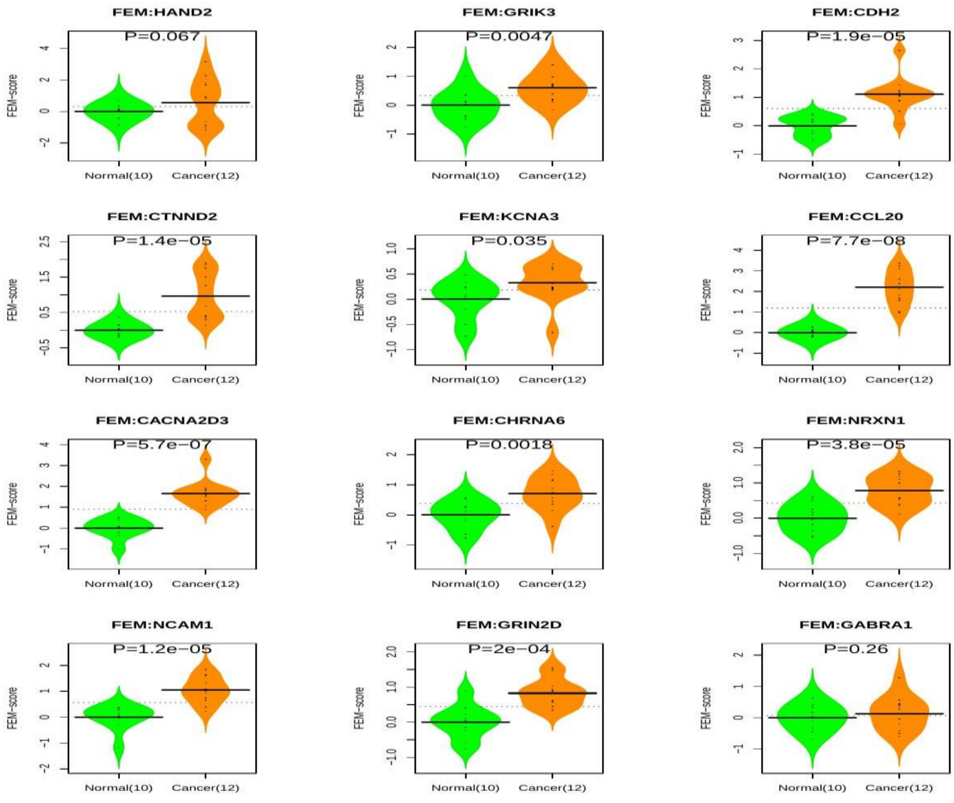

SI fig.S9: Association of FEM-scores in normal-cancer status in GSE92396 gene-expression dataset. Violin plots of FEM-scores for each of the 12 gene-modules. P-value is from a linear model of FEM-score against normal-cancer status with estimated

IC cell fraction as covariate. The relative IC fraction was quantified using GSVA.

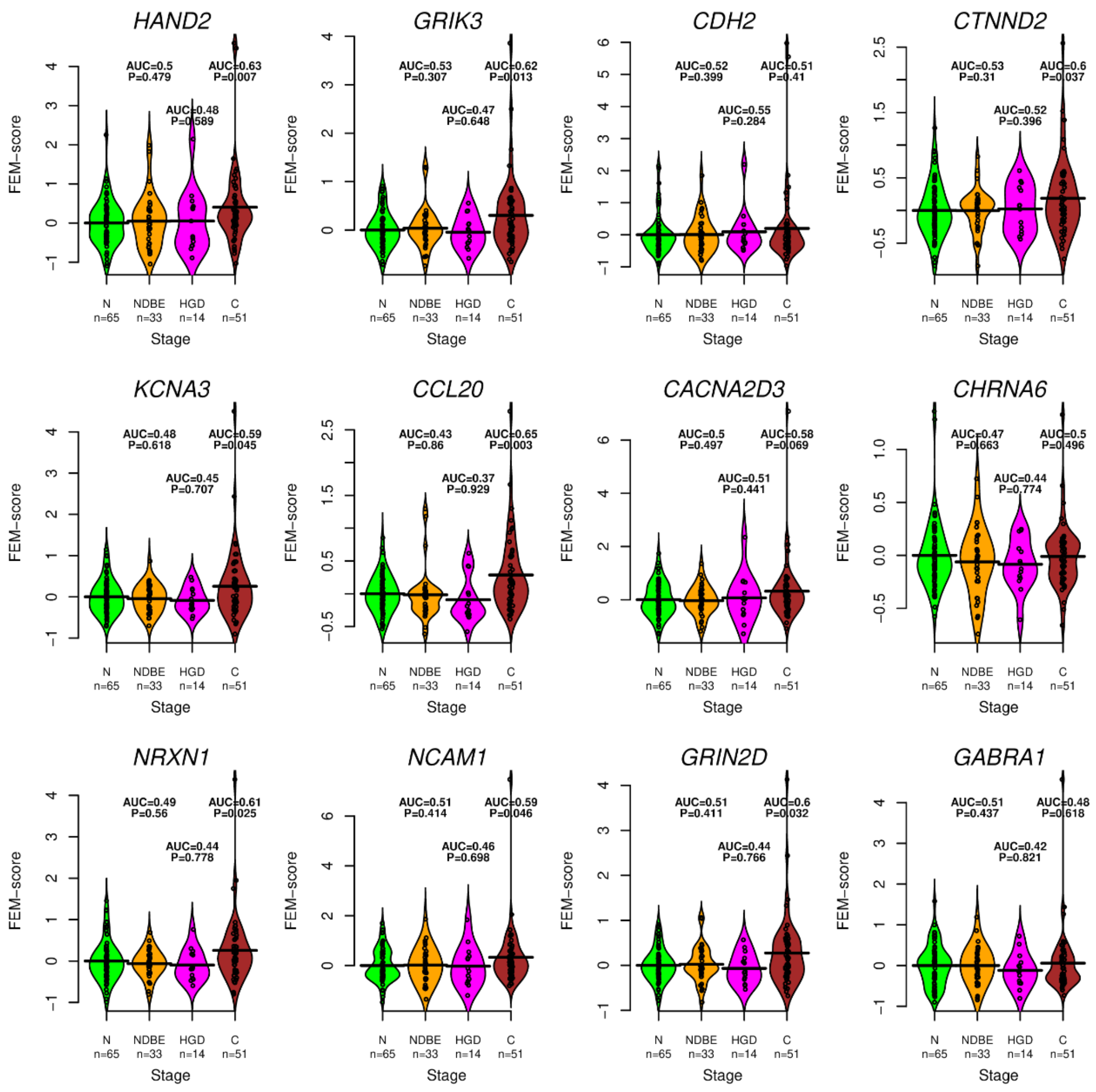

SI fig.S10: Association of FEM-scores in saliva cohort-1. For each of the 12 modules, we display violin plots of the estimated FEM-scores in saliva cohort-1 across the 4 disease stages. N=normal, NDBE=non-dysplastic Barrett's Esophagus, HGD=high-grade dysplasia, C=Esophageal Adenocarcinoma. The number of samples in each stage is given below each violin plot. The AUC and P-values derive from a one-tailed Wilcoxon rank sum-test comparing the NDBE, HGD and C stages to the normal-state

N.

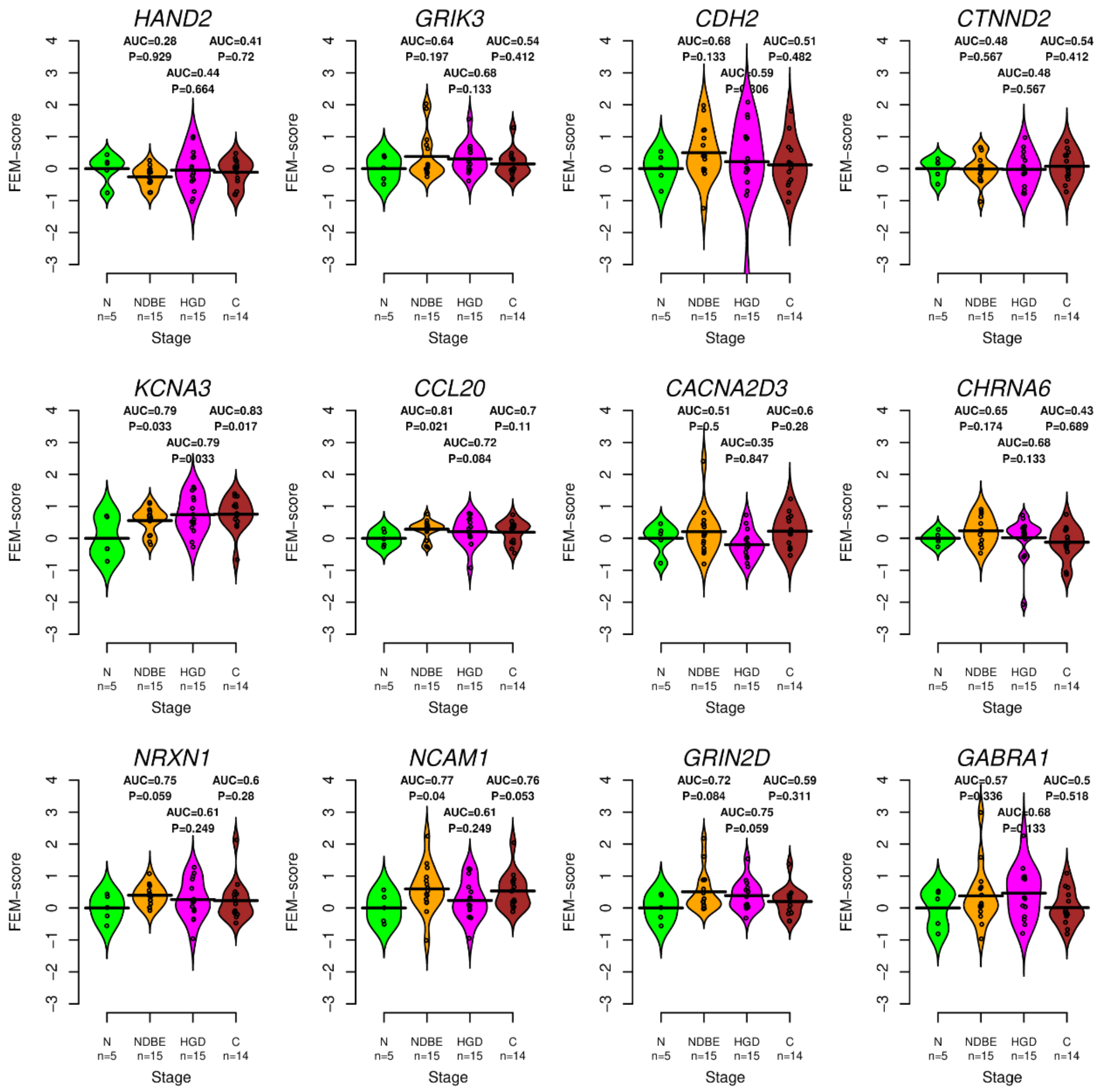

SI fig.S11: Association of FEM-scores in saliva cohort-2. For each of the 12 modules, we display violin plots of the estimated FEM-scores in saliva cohort-2 across the 4 disease stages. N=normal, NDBE=non-dysplastic Barrett's Esophagus, HGD=high-grade dysplasia, C=Esophageal Adenocarcinoma. The number of samples in each stage is given below each violin plot. The AUC and P-values derive from a one-tailed Wilcoxon rank sum-test comparing the NDBE, HGD and C stages to the normal-state N.

SUPPLEMENTARY TABLES

EntrezID (topSeed)	Symbol (topSeed)	Size	Mod	P	Gene
9464	HAND2	10	4.99	0.016	HAND2 CENPB HEYL HAND1 PHOX2A ATF6B SP8 MBTPS2 ZNF519 ZNF273
2899	GRIK3	22	3.75	0.049	GRIK3 GRIN2A DLG3 GRIN1 HAP1 GRIN2B GRIN2C GRIK4 GRIK5 GRIN3A GRIK1 GRIK2 GRIN3B S100A3 RELN LRP8 SHANK1 LIN7B NCALD ADGRL2 SEMA4C PPOX
1000	CDH2	18	6.98	0.003	CDH2 IARS2 MAPK12 CDH4 CDH11 PTPRO BOC CDON ARVCF LRRC7 PTPN22 PTPRG PTPN7 NACA2 TMEM256 NENF PTPRR NTN3
1501	CTNND2	67	4.78	0.001	CTNND2 SPHK1 PCDH7 CDH15 CDH4 CDH10 CDH11 CDH13 CDH16 CDH17 CDH18 CDH19 CDH20 CDH22 CDH23 CDH3 CDH5 CDH6 CDH8 CDH9 CDHR1 CDHR2 CELSR1 CELSR2 CELSR3 DCHS1 FAT3 PCDH10 PCDH11X PCDH11Y PCDH12 PCDH15 PCDH18 PCDH20 PCDH8 PCDHA1 PCDHB10 PCDHB11 PCDHB12 PCDHB13 PCDHB14 PCDHB15 PCDHB16 PCDHB1 PCDHB2 PCDHB3 PCDHB4 PCDHB5 PCDHB6 PCDHB7 PCDHB8 PCDHGA1 LRRC7 ORAI1 CTNNA2 CTNNA3 CTNNAL1 SPHKAP PCDH17 CDON BOC NTN3 PROM1 ATP2C2 CTNNBIP1 P2RX4 MYO7A
3738	KCNA3	11	7.45	0.001	KCNA3 KCNA2 KCNA4 KCNA1 KCNAB3 KCNE4 ETV1 KCNS2 KCNA6 DLGAP1 KCNH7
6364	CCL20	56	3.51	0.018	CCL20 FPR1 CCR5 CXCR4 RALBP1 C5AR1 FPR3 CCR1 CCR3 CCR4 CCR7 CCR8 CCR9 CCRL2 CXCR5 CCL22 CCL8 CCL7 CCL4 CCL3 CCL18 CCL27 CX3CL1 CCL2 CCL26 CCL11 CXCR1 CCL13 CCL5 MMP3 ACKR1 CCL1 SPOCK3 PF4 TPST1 SPOCK2 CMKLR1 TPST2 SPOCK1 MMP1 BCAN MMP13 ADAMTS5 ACAN CMA1 MMP20 HAPLN1 MMP10 MMP7 TNN MMP14 IGFBP3 C5AR2 TNFAIP6 CCL24 MMP19
55799	CACN2D3	12	5.9	0.002	CACNA2D3 CACNB1 CACNA1E CACNG2 CACNG4 CACNA1B CACNB3 CACNB4 CACNA2D2 MRFAP1 RGS12 CACNA2D4
8973	CHRNA6	16	3.87	0.048	CHRNA6 CHRNA7 CHRNA4 CHRNB3 CHRNB2 CHRNB4 CHRNA3 CHRNA5 CHRND CHRNG CHRNA1 CHRNE CHRNA2 CHRNB1 RAPSN

					CHRNA9
9378	NRXN1	15	5.98	0.018	**NRXN1** **SYT9** **NLGN1** **NXPH3** SYTL2 NLGN2 **NLGN3** RPH3A SYT4 SYTL1 NXPH1 **NXPH2** SYT13 **NRXN2** RAB27B
4684	NCAM1	24	5.82	0.001	**NCAM1** CACNA1D **GDNF** **GFRA1** FGF19 NCAN CACNB1 **GFRA2** GFRA4 ARTN CACNB3 CACNB4 NRTN PSPN ST8SIA2 **ST8SIA3** ST8SIA4 **CACNA2D3** CACNA1E CACNG4 **DOK5** **GFRA3** CACNA2D4 TNN
2906	GRIN2D	29	3.9	0.02	GRIN2D SHANK1 GRIN1 HAP1 RASGRF1 CIT **GRIK2** DUSP4 RASGRF2 **GRIK1** **GRIK3** GRIK4 GRIK5 NANOS1 GRIN3A GRIN2C **GRIN2A** GRIN3B DLG3 **S100A3** GRIN2B **RELN** LRP8 PANK2 LIN7B NCALD **ADGRL2** SEMA4C PPOX
2554	GRBRA1	16	4.46	0.021	**GABRA1** GABRB1 GABRB3 GABRA3 GABRA4 **GABRB2** GABRG2 GABRQ GABRA6 **GABRG1** **GABRA5** GABRD ARHGEF9 DGAT1 NEUROD4 LRRN2

SI table.S1: ***FEM gene modules inferred in EAC TCGA cohort.*** *Table lists the Entrez gene ID of the seed-gene, its corresponding gene-symbol, the number of genes in the module, the modularity-score of the module, the P-value of significance against randomization, and the symbols of all module members. Text colors indicate the following: boldface Blue: significant hypermethylation and underexpression, boldface Orange: significant hypomethylation and overexpression, Cyan: significant hypermethylation only, Magenta: significant hypomethylation only, Red: significant overexpression only, Green: significant underexpression only and Black: insignificant differential DNAm and mRNA or differential DNAm and differential mRNA display same direction of effect.*

Data resource	Data Type	Data Histology
GEO(GSE72872)	Illumina Infinium 450K DNA methylation	125 EAC<br>19 Barrett's<br>63 normal adjacent squamous<br>21 normal stomach
GEO(GSE89181)	Illumina Infinium 450K DNA methylation	24 EAC<br>21 BE<br>18 BE+LGD<br>18 BE+GDG<br>11 normal adjacent squamous
GEO(GSE72874)	RNA-seq	48 EAC<br>17 normal
GEO(GSE92396)	Affymetrix array of gene expression	12 EAC<br>10 normal

SI table.S2: ***EAC bulk tissue cohorts used for validation of FEM-modules.*** *Table lists accession numbers, data-type, and the distribution of samples according to histology.*
